# Artificial Cervical Disc Arthroplasty (ACDA): tips and tricks

**Published:** 2012-11

**Authors:** Masoud Khadivi, Vafa Rahimi Movaghar, Sina Abdollahzade

**Affiliations:** ^*a*^Department of Neurosurgery, Shariati Hospital, Tehran university of Medical Sciences, Tehran, Iran.; ^*b*^Sina Trauma and Surgery Research Center, Tehran University of Medical Sciences, Tehran, Iran.

## Abstract

**Background:**

Anterior cervical discectomy and fusion (ACDF) is currently treatment of choice for managing medical therapy refractory cervical degenerative disc disease. Numerous studies have demonstrated the effectiveness of ACDF; patients generally experience rapid recoveries, and dramatic improvement in their pain and quality of life. However, as several studies reported symptomatic adjacent segment disease attributed to fusions’ altered kinematics, cervical disc arthroplasty emerged as a new motion-sparing alternative to fusion. Fusion at one level increases motion at adjacent levels along with increased intradiscal pressures. This phenomenon can result in symptomatic adjacent level degeneration, which can necessitate reoperation at these levels. The era of cervical arthroplasty began in Europe in the late 1990s. In recent years, artificial cervical disc arthroplasty (ACDA) has been increasingly used by spine surgeons for degenerative cervical disc disease. There have been several reports of safety, efficacy and indications of ACDA.

Cervical arthroplasty offers several theoretical advantages over anterior cervical discectomy and fusion (ACDF) in the treatment of selected patients with medically refractory cervical radiculopathy. Preserving motion at the operated level, cervical TDR has the potential to decrease the occurrence of adjacent segment degeneration.

There are a few studies on the efficacy and effectiveness of ACDA compared to cervical fusion. However, the true scenery of cervical arthroplasty yet to be identified.

**Objective:**

This study is intended to define patients' characteristics and outcomes of ACDA by a single surgeon in Iran.

**Methods:**

This retrospective study was performed in two general Hospitals in Tehran, Iran from 2005 To 2010. All patients were operated by one senior neurospine surgeon. One hundred fifty three patients were operated in this period. All patients signed the informed consent form prior to surgery. All patients presented with cervical discopathy who had myelopathy or radiculopathy and failed conservative management, undergoing cervical disc arthroplasty by ACDA were included, consecutively. Patients were followed for at least 2 years.

Exclusion criteria was age greater than 60 years, non compliance with the study protocol, osteoporosis, infection, congenital or post traumatic deformity, malignancy metabolic bone disease, and narrow cervical canal (less than 12 mm). Heterotopic ossification and adjacent segment degenerative changes were assessed at 2 years follow up by means of neutral and dynamic xrays and CT/MRI if clinically indicated. Neck and upper extremity pain were assessed before the procedure and in the first post-operative visit and 3 months later by means of visual analogue scale.

A standard approach was performed to the anterior cervical spine. Patients were positioned supine while holding neck in neutral position. A combination of sharp and blunt dissection was performed to expose longus coli musculature and anterior cervical vertebrae. Trachea and esophagus were retracted medially and carotid artery and jugular vein laterally. After a thorough discectomy, the intersomatic space is distracted in a parallel way by a vertebral distracter. Followed by Caspar distractor is applied to provide a working channel into posterior disc space. In this stage, any remnant disc materials as well as osteophytes are removed and foraminal decompression is done. Posterior longitudinal ligament (PLL) opening and removal, although discouraged by some, is done next. In order to define the size of the prosthesis, multiple trials are tested. It is important not to exceed the height of the healthy adjacent disc to avoid facet joint overdistraction. An specific insertor is applied to plant the prosthesis in disc space. Control X-rays are advised to check the precise positioning of the implant.

**Results:**

one hundred-fifty three patients including 87 females and 66 males were included. The mean age was 41 for females and 42 for males. Affected level was C5-C6 in 81 cases, C6-C7 in 72 cases and C4-C5 in 10 cases. The most common applied ACDA was DiscoCerv which was inserted in 127 cases followed by prodisc-c in three patients and Baguera in thirty three psatients.Ten cases had two levels involvement. Both neck and upper extremity pain improved significantly in early and late post op assessments compared to pre-op. There was only one operative complication of quadriparesis which might be attributed to the iatrogenic cervical spinal trauma.

**Conclusions:**

Cervical disc arthroplasty has been advocated to address drawbacks of fusion including loss of motion segment and adjacent level degeneration; our study along with several other reports provide considerable evidence in this regard. Cervical disc arthroplasty is a safe and effective alternative for fusion in cervical degenerative disc disease.

**Keywords:**

Cervical degenerative disc disease, Artificial cervical disc arthroplasty, Safety, Efficacy


					Volume 4, Suppl. 1 Nov 2012
Publisher: Kermanshah University of Medical Sciences
URL: http://www.jivresearch.org
Copyright:
Congress of Iranian Neurosurgeons, 14-16 November 2012, Kermanshah, Iran
Paper No. 36
Artificial Cervical Disc Arthroplasty (ACDA): tips and tricks
Masoud Khadivi a,*
, Vafa Rahimi Movaghar b
, Sina Abdollahzade a
a
Department of Neurosurgery, Shariati Hospital, Tehran university of Medical Sciences, Tehran, Iran.
b
Sina Trauma and Surgery Research Center, Tehran University of Medical Sciences, Tehran, Iran.
Abstract:
Background: Anterior cervical discectomy and fusion (ACDF) is currently treatment of choice for managing medical
therapy refractory cervical degenerative disc disease. Numerous studies have demonstrated the effectiveness of
ACDF; patients generally experience rapid recoveries, and dramatic improvement in their pain and quality of life.
However, as several studies reported symptomatic adjacent segment disease attributed to fusions' altered
kinematics, cervical disc arthroplasty emerged as a new motion-sparing alternative to fusion. Fusion at one level
increases motion at adjacent levels along with increased intradiscal pressures. This phenomenon can result in
symptomatic adjacent level degeneration, which can necessitate reoperation at these levels. The era of cervical
arthroplasty began in Europe in the late 1990s. In recent years, artificial cervical disc arthroplasty (ACDA) has been
increasingly used by spine surgeons for degenerative cervical disc disease. There have been several reports of
safety, efficacy and indications of ACDA.
Cervical arthroplasty offers several theoretical advantages over anterior cervical discectomy and fusion (ACDF) in
the treatment of selected patients with medically refractory cervical radiculopathy. Preserving motion at the
operated level, cervical TDR has the potential to decrease the occurrence of adjacent segment degeneration.
There are a few studies on the efficacy and effectiveness of ACDA compared to cervical fusion. However, the true
scenery of cervical arthroplasty yet to be identified.
Objective: This study is intended to define patients' characteristics and outcomes of ACDA by a single surgeon in Iran.
Methods: This retrospective study was performed in two general Hospitals in Tehran, Iran from 2005 To 2010. All
patients were operated by one senior neurospine surgeon. One hundred fifty three patients were operated in this
period. All patients signed the informed consent form prior to surgery.
All patients presented with cervical discopathy who had myelopathy or radiculopathy and failed conservative
management, undergoing cervical disc arthroplasty by ACDA were included, consecutively. Patients were followed
for at least 2 years.
Exclusion criteria was age greater than 60 years, non compliance with the study protocol, osteoporosis, infection,
congenital or post traumatic deformity, malignancy metabolic bone disease, and narrow cervical canal (less than 12
mm). Heterotopic ossification and adjacent segment degenerative changes were assessed at 2 years follow up by
means of neutral and dynamic xrays and CT/MRI if clinically indicated.
Neck and upper extremity pain were assessed before the procedure and in the first post-operative visit and 3
months later by means of visual analogue scale.
A standard approach was performed to the anterior cervical spine. Patients were positioned supine while holding
neck in neutral position. A combination of sharp and blunt dissection was performed to expose longus coli musculature
and anterior cervical vertebrae. Trachea and esophagus were retracted medially and carotid artery and jugular
vein laterally. After a thorough discectomy, the intersomatic space is distracted in a parallel way by a vertebral
distracter. Followed by Caspar distractor is applied to provide a working channel into posterior disc space. In this
stage, any remnant disc materials as well as osteophytes are removed and foraminal decompression is done.
Posterior longitudinal ligament (PLL) opening and removal, although discouraged by some, is done next. In order to
Volume 4, Suppl. 1 Nov 2012
Publisher: Kermanshah University of Medical Sciences
URL: http://www.jivresearch.org
Copyright:
Congress of Iranian Neurosurgeons, 14-16 November 2012, Kermanshah, Iran
define the size of the prosthesis, multiple trials are tested. It is important not to exceed the height of the healthy
adjacent disc to avoid facet joint overdistraction. An specific insertor is applied to plant the prosthesis in disc space.
Control X-rays are advised to check the precise positioning of the implant.
Results: one hundred-fifty three patients including 87 females and 66 males were included. The mean age was 41
for females and 42 for males. Affected level was C5-C6 in 81 cases, C6-C7 in 72 cases and C4-C5 in 10 cases. The
most common applied ACDA was DiscoCerv which was inserted in 127 cases followed by prodisc-c in three patients
and Baguera in thirty three psatients.Ten cases had two levels involvement. Both neck and upper extremity pain
improved significantly in early and late post op assessments compared to pre-op. There was only one operative
complication of quadriparesis which might be attributed to the iatrogenic cervical spinal trauma.
Conclusion: Cervical disc arthroplasty has been advocated to address drawbacks of fusion including loss of motion
segment and adjacent level degeneration; our study along with several other reports provide considerable evidence
in this regard. Cervical disc arthroplasty is a safe and effective alternative for fusion in cervical degenerative disc
disease.
Keywords:
Cervical degenerative disc disease, Artificial cervical disc arthroplasty, Safety, Efficacy
* Corresponding Author at:
Masoud Khadivi: MD, Department of Neurosurgery, Shariati Hospital, Tehran university of Medical Sciences, Tehran, Iran.
Tel/Fax: +98 21 88220040, Email: maskhadivi.dr@gmail.com, (Khadivi M.).

				

